# Increased Drug Resistance and Biofilm Formation Ability in ST34-Type *Salmonella* Typhimurium Exhibiting Multicellular Behavior in China

**DOI:** 10.3389/fmicb.2022.876500

**Published:** 2022-03-18

**Authors:** Kaifeng Chen, Yuan Gao, Lili Li, Weixiao Zhang, Jiayi Li, Zhouping Zhou, Haishan He, Zeluan Chen, Ming Liao, Jianmin Zhang

**Affiliations:** ^1^Center of Emerging and Zoonotic Diseases, National and Regional Joint Engineering Laboratory for Medicament of Zoonoses Prevention and Control, Key Laboratory of Zoonoses, Ministry of Agriculture, Key Laboratory of Zoonoses Prevention and Control of Guangdong Province, Guangdong Laboratory for Lingnan Modern Agriculture, College of Veterinary Medicine, South China Agricultural University, Guangzhou, China; ^2^Institute of Animal Health, Guangdong Academy of Agricultural Sciences, Guangzhou, China

**Keywords:** *S.* Typhimurium, animal source food, ST34, multicellular behavior, biofilm, antibiotic resistance

## Abstract

*Salmonella* Typhimurium is an important food-borne pathogen. In this paper, multicellular behavior and associated characteristics of *S.* Typhimurium isolated from human and animal source food were studied. All the *S.* Typhimurium strains exhibiting multicellular behavior (100%) belonged to the ST34 type. In addition, most of the ST34-type multicellular behavior *S.* Typhimurium strains had a human origin (69.11%) and 98% of the ST34-type multicellular behavior strains exhibited strong biofilm formation capacity, which was much higher than that of non-multicellular behavior strains (7%, *P* < 0.01). Antibiotic resistance in ST34-type multicellular behavior strains was significantly higher than in strains with non-multicellular behavior for most conventional drugs (*P* < 0.05); notably, Polymyxin B (8%) and Imipenem (1%) resistances were also observed in the ST34-type strains. Furthermore, all the ST34-type multicellular behavior strains (100%) exhibited Multiple Drug Resistance (resistance to ≥3antibiotics), which was much higher than that of the non-multicellular behavior strains (*P* < 0.05). Consistent with the drug-resistant phenotype, the carrying rates of most drug-resistant genes in ST34-type multicellular behavior strains were higher than that those in non-multicellular behavior strains (*P* < 0.05). Therefore, this study revealed the emergence of a prevalent ST34-type multicellular behavior *S.* Typhimurium strains with increased biofilm formation ability and drug resistance rate, which poses a threat to public health safety, and highlights the need for comprehensive monitoring of the strains.

## Introduction

*Salmonella* is an important zoonotic pathogen that causes food-borne diseases in many countries (Gomez et al., 1997). *Salmonella* has been reported to be the leading cause of death among food-borne bacterial pathogens in some countries (Casey et al., 2011) with 94 million infections annually and 155,000 deaths globally (Hendriksen et al., 2011). *S.* Typhimurium is one of the dominant serotypes among the >2,500 recognized *Salmonella* serotypes (Popoff et al., 2001; Liang et al., 2015; Yang et al., 2017). Furthermore, *S.* Typhimurium has numerous hosts and is widely distributed; it can not only infect animals but is also transmitted to humans through contaminated food (Adagbada et al., 2014) and the mortality caused by *S.* Typhimurium is 3-fold higher than the average mortality associated with *Salmonella* (Angulo and Kre, 2005).

*S.* Typhimurium has different sequence types (STs), which exhibit distinct biological characteristics including virulence (Singletary et al., 2016) and drug resistance (Li et al., 2017). An analysis of *S.* Typhimurium strains in the Enterobase database^1^ revealed that from the end of the 19th century to the end of the 1980s, all the *S.* Typhimurium isolates belonged to ST19; however, since 1990, the proportion of ST34 has increased annually and has reportedly replaced ST19 in recent years as the prevalent ST (Wong et al., 2013; Li et al., 2017). Consequently, the prevalence trends of *S.* Typhimurium STs should be monitored constantly to facilitate the effective prevention and control of the associated epidemics.

Currently, the treatment of diseases related to *S.* Typhimurium infection mainly involves the administration of antibiotics. However, in the wake of widespread antibiotic use, the rapid emergence of Multi-Drug Resistance (MDR) is a new challenge for the global prevention and control of *S.* Typhimurium infection. *S.* Typhimurium can exhibit multicellular behavior under drug or environmental pressure (Römling, 2001) and the prevalence of *S.* Typhimurium exhibiting multicellular behavior has been reported sporadically in Ethiopia (Eguale et al., 2014) and Germany (Cimdins and Simm, 2017a), among other countries. Multicellular behavior of *S.* Typhimurium refers to the transition of strains from a single-cell planktonic state to a quiescent aggregated colony state and as a community (Römling, 2001), and studies have demonstrated that bacteria with multicellular behavior are mainly characterized by production of extracellular matrix in the forms of curli fibers and cellulose (Rmling et al., 1998; Zogaj et al., 2010), which can be combined with diazodye Congo Red so that they can show the Rdar (red, dry, and rough) phenotype on the Congo Red plate. On the contrary, those strains that are unable to produce multicellular behavior are usually characterized by saw (smooth and white) phenotypic, which do not express these matrix components (Cimdins and Simm, 2017b). Furthermore, in bacterial communities formed by multicellular behavioral strains, the matrix-coated bacteria are considered to have structural protection, which can enhance the strains’ adaptability to adverse external environments and their stress tolerance (Eguale et al., 2014). Consequently, such structural protection poses a challenge to typical strategies of prevention and control of *S.* Typhimurium.

Although *S.* Typhimurium is one of the most prevalent *Salmonella* serotypes in China (Lu et al., 2011), systematic studies on the prevalence and associated roles of multicellular behavior in the increasingly prevalent ST34-type *S.* Typhimurium are still lacking. Monitoring the prevalence of multicellular behavior in *S.* Typhimurium and studying the associated biological characteristics could facilitate *Salmonella* infection prevention and control. Therefore, this study explored the prevalence and associated biological characteristics of multicellular behavior in *S.* Typhimurium isolated from human and animal source food in China. In addition, the authors investigated the factors that could facilitate the prevalence of the ST34-type *S.* Typhimurium and highlight the potential risks posed by multicellular behavior in *S.* Typhimurium to public health and safety.

## Materials and Methods

### Bacterial Strains

*Salmonella* Typhimurium isolates were recovered from samples of clinically suspected patients and animal source food (such as chicken, pork, meat products, and breeding environment) in Fujian, Guangxi, Guangzhou, Hubei, Shandong, Shanxi, Shanghai, Sichuan, Xinjiang, and Chongqing from 2008 to 2017. Among them, 123 strains (61.50%) were of human origin, and 77 strains (38.50%) were of animal food origin. These strains were preserved at the Key Laboratory of Zoonoses Prevention and Control of Guangdong Province, Guangzhou, China.

### Detection of the Multicellular Behavior Morphotype

The identification of multicellular behavioral phenotypes was carried out based on methods in previous reports, with slight modifications (Zhang et al., 2019). Briefly, 10 μl of the strains cultured overnight was dropped on a salt-free LB agar plate containing 40 mg/ml Congo Red and 20 mg/ml Coomassie Brilliant Blue G, and cultured at 27°C for 5 d. Subsequently, colony morphology on the plate was observed to determine whether the strain exhibited multicellular behavior, which was characterized based on the RDAR (red, dry, and rough) phenotype. On the contrary, those strains that were unable to produce multicellular behavior were characterized by saw (smooth and white) phenotypic.

### Multi-Locus Sequence Typing Analysis

The genomic DNA of the bacteria was extracted by the boiling method as previously reported (Rmling et al., 1998); afterward, based on the primers designed by University of Cork (Achtman et al., 2017), seven pairs of housekeeping genes, including, *aroC*, *dnaN*, *hemD*, *hisD*, *purE*, *sucA*, and *thrA* of *Salmonella*, were amplified by PCR, and the PCR products were sequenced and submitted to the Enterobase database^2^ to obtain the corresponding allele numbers. Seven housekeeping genes together constitute the STs of all strains in this study.

### Detection of Biofilm Formation Ability

Biofilm formation ability was assessed using a previously described protocol, with some modifications (Gomez-Baltazar et al., 2019). First, a single colony was added to Lucia-Bertani broth (LB) and cultured overnight at 37°C with shaking. On the following day, the bacterial solution was diluted into fresh salt-free LB broth (1,100), and the diluted bacterial solution (200 μl) from each sample was inoculated into each well of 96-well plates, and 200 μl of salt-free LB broth added to the well as a blank control, then incubated at 27°C for 48 h without shaking. Afterward, the bacterial suspension was discarded and the plates were washed three times with PBS solution. After drying, 200 μl of anhydrous methanol was added to each well and fixed for 15 min. The anhydrous methanol was discarded and the plates were dried; thereafter, 200 μl of 1% crystal violet was added to each well and dyed at 24–26°C for 15 min. Unbound dyes were removed gently and the plates were washed three times with PBS solution. After drying at 24–26°C, 200 μl of 33% acetic acid was added to the well to dissolve crystal violet. Finally, optical density was measured at 595 nm using an enzyme-labeling instrument (BIO-TEK). The strains were classified into four categories: strong biofilm forming ability, medium biofilm forming ability, weak biofilm forming ability, and none biofilm forming ability according to the criteria described in a previous study (Diez-Garda et al., 2012).

### Antimicrobial Susceptibility Tests

Detection of *S.* Typhimurium susceptibility to antimicrobials was carried out using the Agar dilution method as described by the Clinical and Laboratory Standards Institute (CLSI; Cockerill et al., 2012). *Escherichia* coli ATCC25922 was used as the quality control strain. In the present study, 15 antimicrobial agents that accounted for 10 classes of antibiotics were used for the susceptibility analyses. The results of antibiotic sensitivity were assessed according to the CLSI criteria.

### Detection of Antibiotic Resistance Genes

The PCR method was used to detect the resistance genes of isolates with the ACSuT (ampicillin, chloramphenicol, sulfamethoxazole, and tetracyclinen resistance) profile, and the resistance genes of isolates resistant to Polymyxin B and Imipenem. Therefore, PCR screening of 10 resistance genes, including *BlaCTXM*, *BlaTEM*, *Bloxa*, *BlaNDM-5*, *flo*, *sulI*, *sulII*, *TetA*, *TetB*, and *mcr-1*, was carried out (Ahmed et al., 2007; Az et al., 2019; Wang et al., 2019).

### Statistical Analysis

The data were analyzed using GraphPad Prism v8.0 (GraphPad Software, San Diego, CA, United States). The Chi-square test was used to compare two test groups. *p* < 0.05 indicated significant difference.

## Results

### Detection of the Multicellular Behavior Morphotype

**Figure 1:**
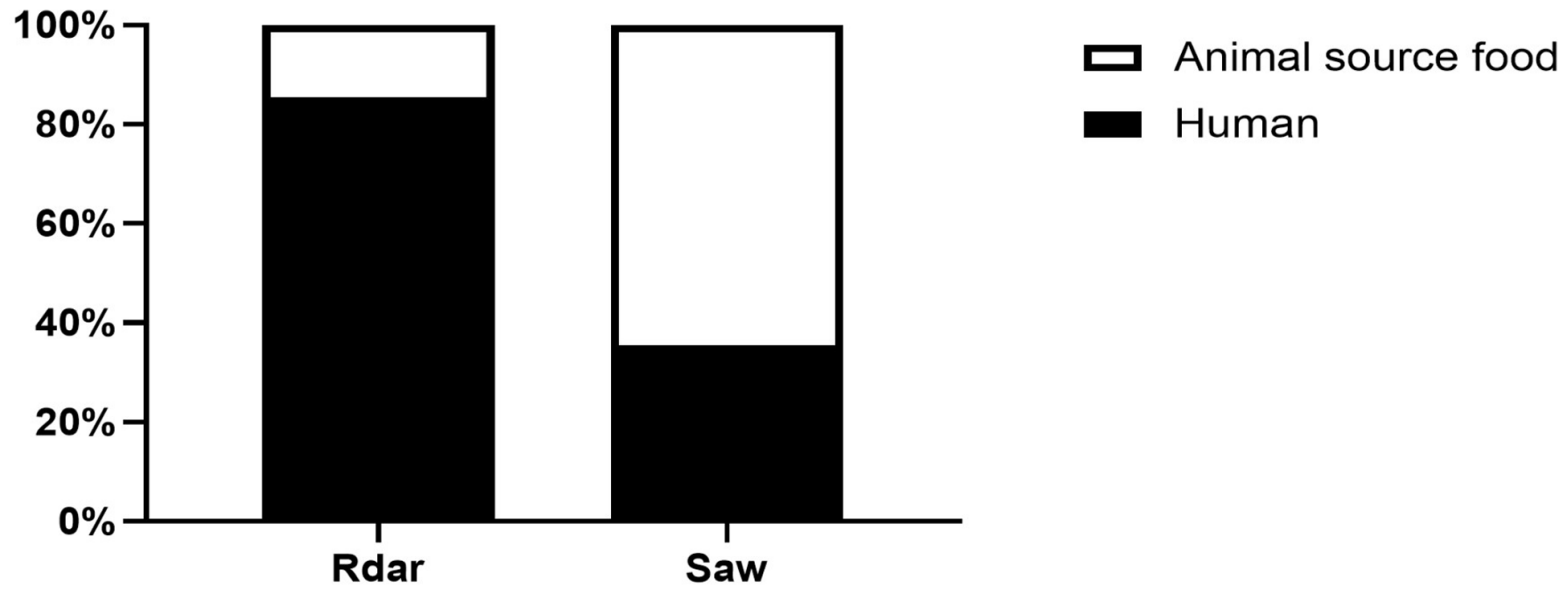
Identification of multicellular behavior phenotypes of *Salmonella* Typhimurium from human- and animal source food.

*S.* Typhimurium with multicellular behavior detection was conducted by counting the strains displaying the Rdar phenotype on Congo red agar plate. Among 123 isolates from human sources, 85 (69.11%) and 38 (30.89%) exhibited the Rdar and saw phenotypes, respectively. Conversely, among the strains isolated from animal sources food, 15 (19.48%) and 62 (80.52%) strains exhibited the Rdar and saw phenotypes, respectively (Figure 1).

### Multi-Locus Sequence Typing Analysis

**Figure 2:**
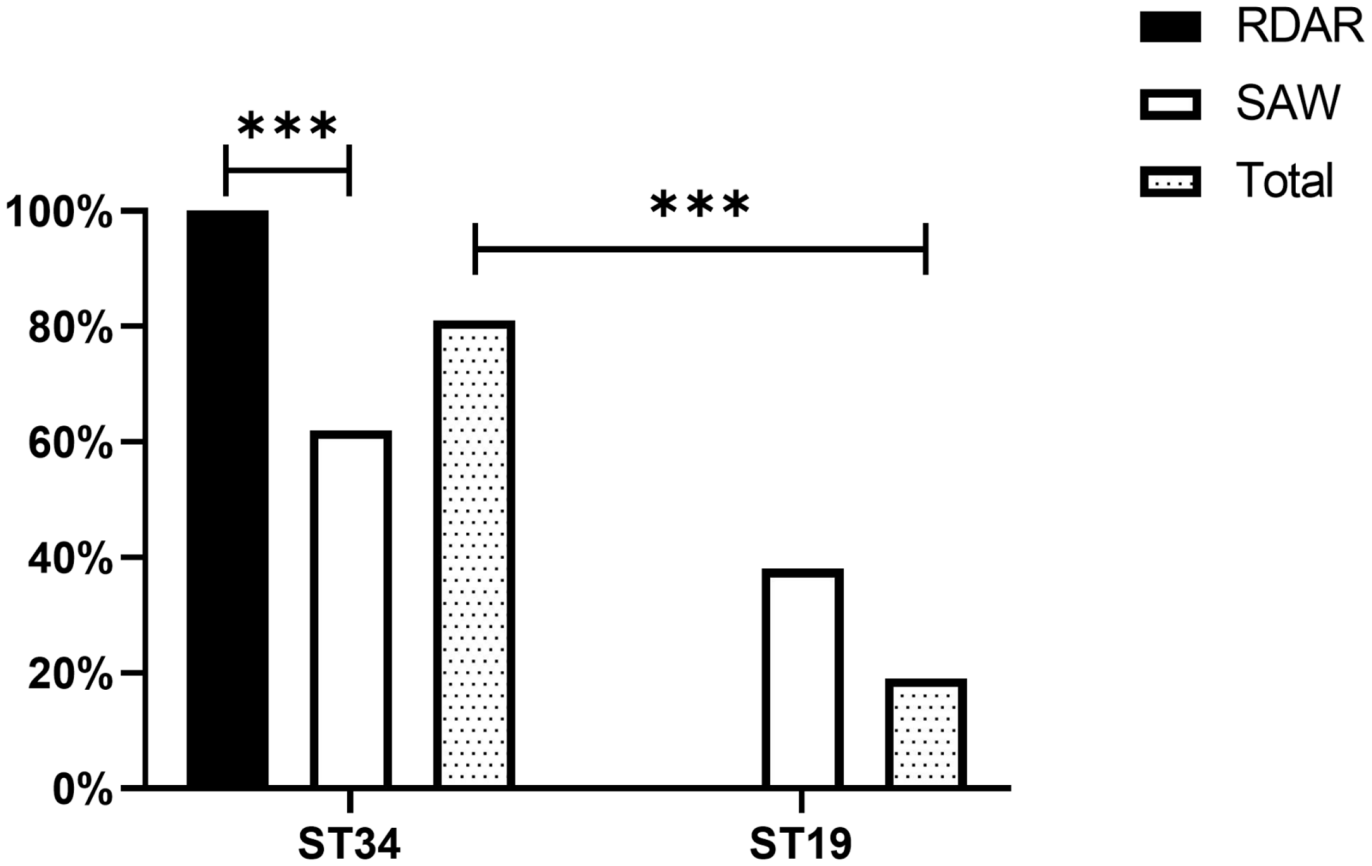
Multi-locus sequence typing of colonies of different phenotypes (***p < 0.001).

A total of 81.00% of the strains detected belonged to the ST34 genotype, whereas only 19.00% of the strains belonged to the ST19 genotype. In addition, there were significant differences in ST typing between the Rdar and saw phenotypes. All the Rdar phenotype colonies (100%) belonged to the ST34 genotype, and all the remaining saw phenotypes (38%) belonged to the ST19 genotype (Figure 2). Between the two sources, 87.80% (108/123) of the strains of human origin belonged to the ST34 genotype, and 70.13% (54/77) of animal food origin belonged to the ST34 genotype (Table 1).

**Table 1:** Multi-locus sequence typing (MLST) of different of *Salmonella* Typhimurium isolates from human- and animal source food.

ST type	Human	Animal-based food
ST34	87.8%(108/123)	70.1%(54/77)
ST19	12.2%(15/123)	29.9%(23/77)

### Biofilm Formation Ability of Colonies of Different Phenotypes

**Figure 3:**
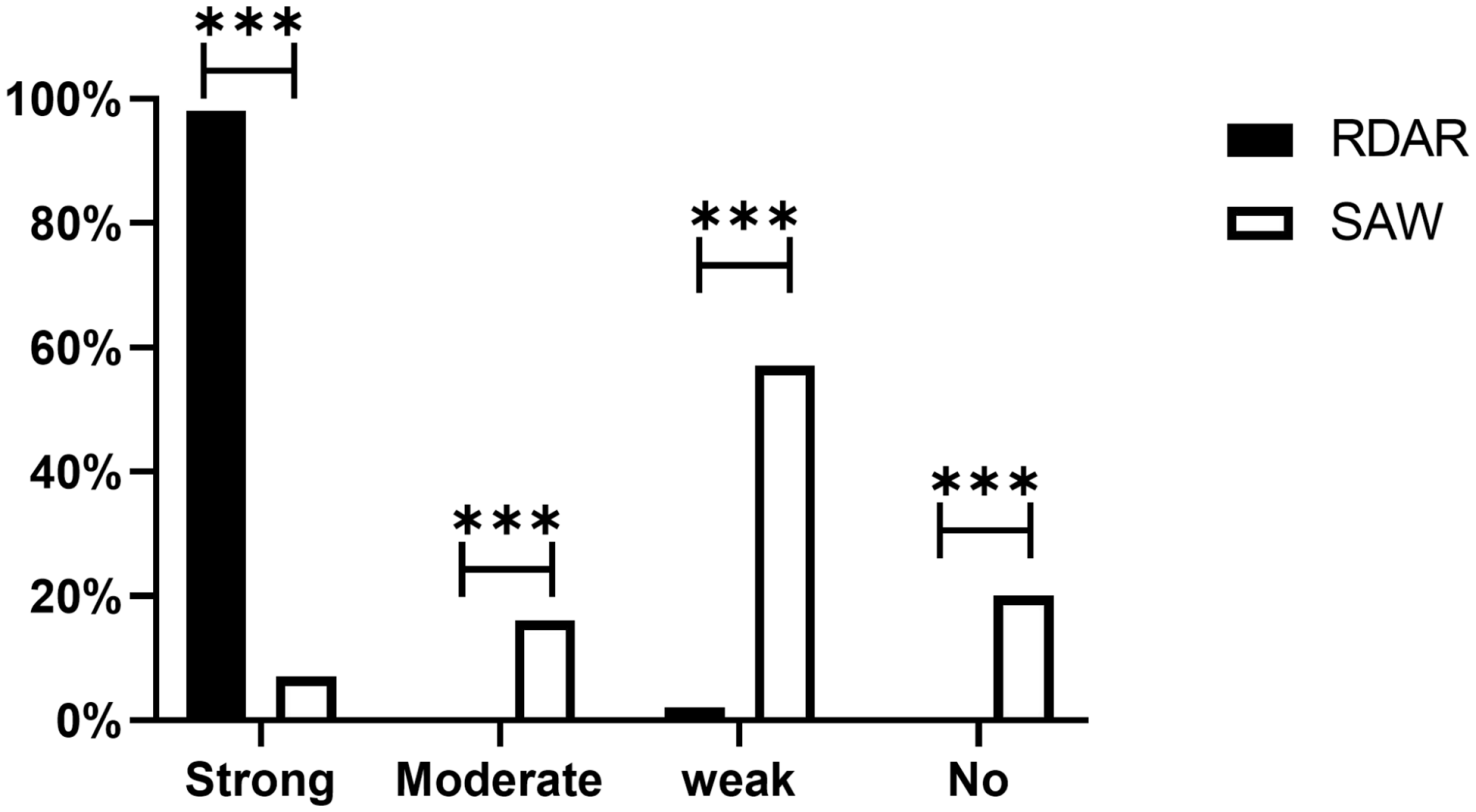
Biofilm formation ability of different phenotype colonies. Strong, Strong biofilm formation ability; Moderate, Moderate biofilm formation ability; Weak, Weak biofilm formation ability; and No, none biofilm formation ability (***p < 0.001).

All the ST34-type multicellular behavior *S.* Typhimurium had the ability to form biofilms, and 98% exhibited strong biofilm formation ability. However, only 7% of the non-multicellular behavior *S.* Typhimurium exhibited strong biofilm formation ability. Among the non-multicellular behavior *S.* Typhimurium, most (57%) showed weak biofilm formation ability, and 16% of the non-multicellular behavior strains exhibited moderate biofilm formation ability. Furthermore, all strains with no biofilm formation ability were non-multicellular behavior (Figure 3). Chi-square test results showed that strong biofilm formation ability was significantly more prevalent in Rdar phenotypes than in saw phenotypes (*P* < 0.01).

### Antimicrobial Susceptibility

**Figure 4:**
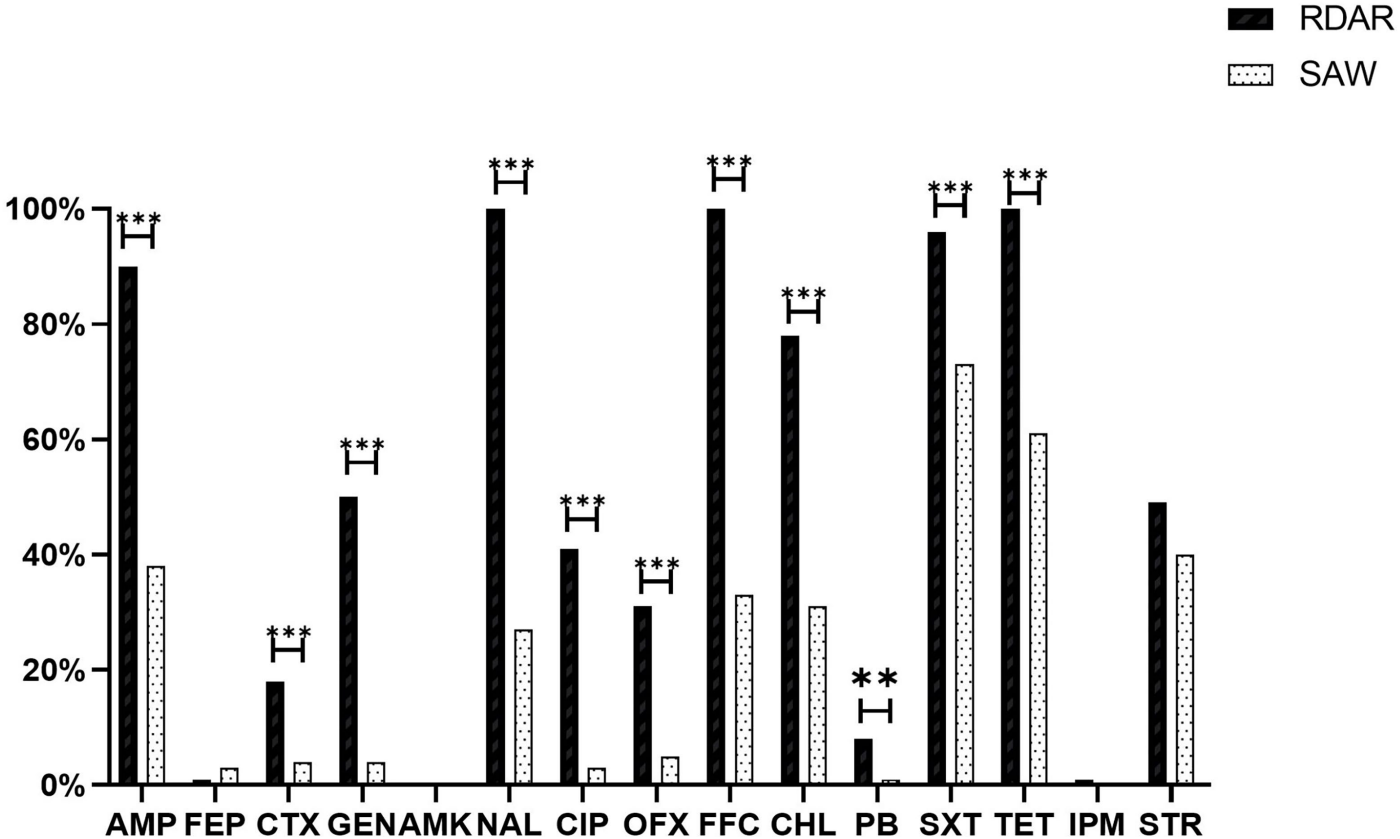
Antimicrobial resistance of isolates and different phenotype colonies. AMP, Ampicillin; FEP, Cefepime; CTX, Cefotaxime; GEN, Gentamicin; AMK, Amikacin; NAL, Nalidixic acid; CIP, Ciprofloxacin; OFX, Ofloxacin; FFC, Florfenicol; CHL, Chloramphenicol; PB, Polymyxin B; SXT, Sulfaisoxazole; TET, Tetracycline; IPM, Imipenem; and STR, Streptomycin (**p < 0.01 and ***p < 0.001).

Among all the strains, resistance to Sulfisoxazole (84.50%) was the most frequently observed, followed by resistance to Tetracycline (80.50%), Florfenicol (66.50%), Ampicillin (64.00%), and Nalidixic acid (63.50%). However, resistance to Cefepime (2.00%) and Amikacin (0%) was low. Additionally, ST34-type multicellular behavior *S.* Typhimurium exhibited greater levels of resistance than non-multicellular behavior *S.* Typhimurium, with significant differences in antibiotic resistance, based on the Chi-square test (*p* ≤ 0.05; Figure 4). Notably, eight out of the nine strains resistant to Polymyxin B were ST34-type multicellular behavior strains. In addition, one strain resistant to Imipenem was an ST34-type multicellular behavior strain.

**Figure 5:**
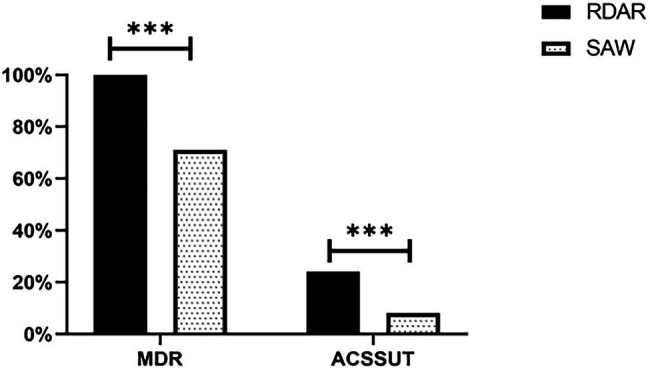
Distribution of multi-drug resistance (MDR) in isolates and their ACSSuT profiles. MDR, Multiple antibiotic resistance; ACSSuT, resistance to Ampicillin, Chloramphenicol, Streptomycin, Sulfamethoxazole, and Tetracycline (***p < 0.001).

Among all the strains, 85.50% developed Multi-Drug Resistance (MDR), and all the ST34-type multicellular behavior strains (100%) were phenotypically resistant to at least three classes of antimicrobial agents. A total of 32 (16.0%) isolates exhibited an ACSSuT resistance profile, and among the ST34-type multicellular behavior strains, 24% exhibited the ACSSuT profile, which was significantly higher than the rate observed for the non-multicellular behavior strains (8%; *P* < 0.01; Figure 5).

### Antibiotic Resistance Genes

The ACSuT resistance genes included *BlaCTXM* (8.50%), *BlaTEM* (44.00%), *Blaoxa* (32.50%), *Flo* (44.00%), *Sul1* (27.00%), *Sul2* (66.00%), *TetA* (11.50%), and *TetB* (67.50%). In addition, one imipenem-resistant strain was found to harbor a *BlaNDM-5*-carrying IncX3 plasmid. The strain also had a IncHI2 plasmid that harbored nine resistant genes, including *aadA1*, *aadA3*, *aph(3′)-la*, *sul1*, *sul2*, *sul3*, *floR*, *cmlA*, and *dfrA12*. Moreover, all the Polymyxin B-resistant strains (100%) harbored the *mcr-1* resistance gene.

**Figure 6:**
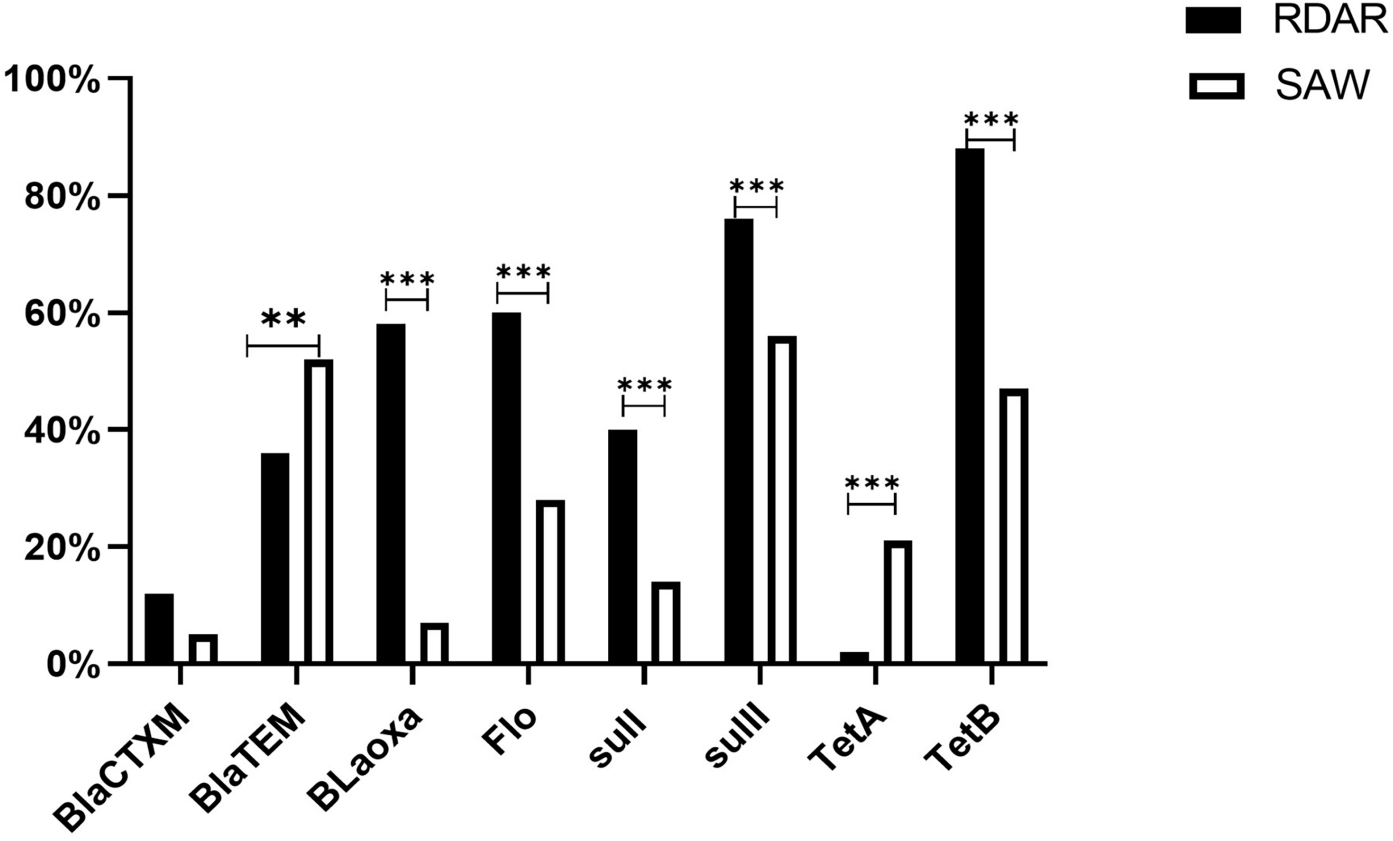
Antimicrobial resistance genes of ACSuT, Ampicillin, Chloramphenicol, Sulfamethoxazole, and Tetracycline. Among them, *BlaCTXM, BlaTEM, and Bloxa* are β-lactam resistance genes, *Flo* is Phenicols resistance genes, *sulI* and *sulII* are Sulphonamide resistance genes, *TetA* and *TetB* are Tetracycline resistance genes, *mcr-1* is the Polymyxin B resistance gene, and *BlaNDM-5* is the Imipenem resistance gene (**p < 0.01 and ***p < 0.001).

Analysis of the resistance genes of different phenotypes revealed that excluding *BlaTEM* and *TetA*, the detection rates of other antibiotic-resistant genes were higher in the ST34-type strains exhibiting multicellular behavior than in the non-multicellular strains (Figure 6). In addition, there were significant differences in the resistance genes between the Rdar and saw morphotypes (*P* < 0.01, Figure 6). Therefore, the resistance gene carrying rates of the ST34-type multicellular behavior strains were much higher.

## Discussion

*S.* Typhimurium is an important food-borne pathogen. It not only causes economic losses in the livestock and poultry breeding industry, but also poses serious risks to human health (Diez-Garda et al., 2012). The results of the present study revealed that ST34 is currently the main *S.* Typhimurium ST type from human and animal source food in China. Other studies have also reported that ST34 has replaced ST19 as the main ST type in some regions in China (Li et al., 2017) and in some other countries (Biswas et al., 2019). To explore the biological characteristics of the emerging ST34 epidemics, and the underlying factors involved in its prevalence and spread, we analyzed the capacities of different strains to exhibit multicellular behavior and their tolerance to antimicrobial factors. Generally, all the *S.* Typhimurium strains exhibiting multicellular behavior (100%) belonged to the ST34 type. Compared to other strains, the strains exhibiting multicellular behavior could have greater environmental adaptability and higher antibiotic resistance. Consequently, the multicellular behavior in the strains could be facilitating the gradual increase in the prevalence of the ST34-type strains.

Notably, according to the results of the present study, most of the ST34-type multicellular behavior strains were of human origin, and the finding is consistent with the results of Vestby et al. (Ahmed et al., 2007). In addition, Römling et al. (2003) reported that human-derived *S.* Typhimurium mainly exhibits multicellular behavior. Human-derived *S.* Typhimurium is more likely to exhibit multicellular behavior, which may be linked to the environments of the strains (Wang et al., 2019). Better or stronger antibiotics would be used to prevent and control *S.* Typhimurium in clinical practice, which puts human-derived *S.* Typhimurium under greater survival pressure, which could in turn facilitate the emergence of multicellular behavior and development of adaptive resistance (Biswas et al., 2019).

In the present study, some *S.* Typhimurium strains from different sources (human origin and animal source food origin) were observed to belong to the same ST type, and most human-derived *S.* Typhimurium strains exhibited multicellular behavior, which highlights the potential role of multicellular behavior in facilitating *S.* Typhimurium spread between animals and humans, and further, in epidemics. Generally, the emergence of multicellular behavior may be the reason for the current high prevalence of ST34, so that further research on the adaptability of strains exhibiting multicellular behavior to unfavorable environments, based on biofilm formation and drug resistance characteristics, should be carried out, which could enhance the capacity of clinicians to manage the threats posed by such strains to public health.

In the present study, we observed that all ST34-type multicellular behavior *S.* Typhimurium isolates could form biofilms, and most of them (98%) displayed strong biofilm formation ability. The proportion observed was much higher than the proportion reported previously (24.14%; Manafi et al., 2020). On the contrary, most of the non-multicellular behavior strains in this study exhibited weak or no biofilm formation ability, indicating that ST34-type multicellular behavior *S.* Typhimurium had stronger biofilm formation ability. This may be due to the strains exhibiting multicellular behavior being characterized by the production of extracellular matrix, in the form of cellulose and curli, which can facilitate the establishment of hydrophobic networks in colonies and enhance biofilm formation (White et al., 2006). Therefore, the results of the present study reveal that the ST34-type multicellular behavior *S.* Typhimurium has a high capacity to form biofilms, which could enhance its adaptability to unfavorable environments and resistance to environmental stress, including antimicrobials (Farzan et al., 2008). The ability of *S.* Typhimurium to form biofilms increases the risk of outbreaks, because such strains can be released from the biofilms and cause food contamination during processing.

According to the results of the present study, the ST34-type multicellular behavior strains exhibited higher drug resistance than non-multicellular behavior strains. The resistance rates of ST34-type multicellular behavior strains to Nalidixic acid, Florfenicol, and Tetracycline were as high 100%, and the resistance rates to drugs such as Ampicillin exceeded 90%, which were higher than the previously reported resistance rates to Nalidixic acid (28.8%; Lina et al., 2018), Florfenicol (93%; Farzan et al., 2008), Tetracycline (68%; Li et al., 2017), and Ampicillin (72.2%; Tu et al., 2015). Notably, all of the ST34-type multicellular behavior strains (100%) in the present study displayed MDR, which is much higher than the previously reported MDR rate in *S.* Typhimurium (56.58%; Ke et al., 2014) and in the non-multicellular behavior *S.* Typhimurium in the present study. The ACSSuT profile is an important indicator for evaluating the drug resistance of non-typhoid *Salmonella*. In the present study, 24% of ST34-type multicellular behavior *S.* Typhimurium exhibited the ACSSuT resistance profile, which was a much higher rate than that of non-multicellular behavior *S.* Typhimurium, and higher than a 14.5% rate reported previously (Ma et al., 2018). The higher drug resistance of the ST34-type multicellular behavior strains could be attributed to excessive and improper use of antibiotics in clinical and animal breeding settings, which could lead to the adaptation of multicellular behavior in *S.* Typhimurium in response to stress. Furthermore, the extracellular matrix produced by strains with multicellular behavior forms a complex network structure that wrap the strains, and the ensuing protective mechanism could enhance resistance of the strains to antibiotics, so that the emergence of ST34-type multicellular behavior *S.* Typhimurium presents additional challenges for its prevention and control.

Considering the high drug resistance and MDR rates of the ST34-type multicellular behavior *S.* Typhimurium, the authors further studied the drug resistance characteristics of the ST34-type multicellular behavior *S.* Typhimurium at the molecular level. In general, the detection rates of drug-resistant genes in the ST34-type multicellular behavior *S.* Typhimurium were higher than those in the non-multicellular behavior strains. The trend could be attributed to the colonies formed by multicellular behavior strains offering opportunities for intra- and inter-species genetic exchange of antibiotic resistance genes (Diez-Garda et al., 2012; Eguale et al., 2014), which facilitates the emergence and spread of antibiotic resistance.

Contrary to expectations, although *blaTEM* is the main gene detected in β-lactamase resistance genes, its detection rates in non-multicellular behavioral phenotypic strains were much higher than in the ST34-type multicellular strains. Similarly, *TetA* had a higher detection rate in non-multicellular behavior strains for tetracycline antibiotic resistance genes. Overall, the results demonstrate why the multicellular behavior *S.* Typhimurium has high drug resistance, in addition to more drug resistance genes, multicellular behavior could facilitate the development of drug resistance, and further in-depth research and analyses are required on the underlying mechanisms of drug resistance. In addition, novel antimicrobial agents should be developed based on a multicellular behavior perspective.

The ST34-type multicellular behavior strains not only exhibited high drug resistance to conventional drugs but also to the last line of defense, Polymyxin B (8%) and Imipenem (1%). The rates were much higher than the resistance rates to Polymyxin B (2.0%) and Imipenem (0.5%) reported previously in China (Lina et al., 2018). The imipenem-resistant strains observed in the present study were isolated from animal source food rather than human sources. Therefore, the multicellular behavior of the strains is a source of concern. Furthermore, in the present study, all (100%) strains resistant to Polymyxin B harbored the *mcr-1* resistance gene. Plasmid-mediated *mcr-1* can be transferred horizontally between different strains (Liu Liu et al., 2016) and is considered a novel “super bacteria” after *NDM-1*; the high detection rate of *mcr-1* in ST34-type multicellular behavior strains may be due to the presence of the gene in the strains with multicellular behavior that are coated with various substrates, which is conducive for the survival and transfer of host strains. In addition, in the present study, the Imipenem-resistant ST34-type multicellular strain harbored a *BlaNDM-5*-carrying IncX3 plasmid. In addition, the strain has been reported to harbor an IncHI2 plasmid that carries nine resistance genes, including *aadA1*, *aadA3*, *aph(3′)-la*, *sul1*, *sul2*, *sul3*, *floR*, *cmlA*, and *dfrA12* (Gao et al., 2020). Therefore, the emergence of an Imipenem-resistant ST34-type multicellular strain has implications for the emergence and spread of drug resistance. The findings also suggest that compared with non-multicellular behavioral strains, the ST34-type multicellular behavioral strains isolated in the present study have stronger drug resistance, with resistance against the last line of defense, which makes the prevention and control of *S.* Typhimurium more challenging.

In summary, multicellular behavior is potentially a major factor involved in the MDR of *S.* Typhimurium. Therefore, strategies for the prevention and control of *S.* Typhimurium should consider whether the strains exhibit a multicellular behavior phenotype.

## Conclusion

In summary, the present study observed the emergence of ST34-type multicellular behavior *S.* Typhimurium isolated from different sources, including human and animal source food, in China. All the ST34-type multicellular behavior *S.* Typhimurium (100%) could form biofilms and 98% exhibited strong biofilm formation ability. In the drug resistance tests of the strains, the ST34-type multicellular behavior strains not only exhibited higher resistance than non-multicellular behavior strains under conventional antimicrobial drugs, but also was resistant to the last line of defense, Polymyxin B (8%) and Imipenem (1%). Furthermore, the ST34-type multicellular strains exhibited much higher MDR rates than the non-multicellular behavior strains and harbored more drug-resistant genes. The emergence of an ST34-type multicellular behavior *S.* Typhimurium with high biofilm formation ability and high drug resistance in China could facilitate the prevalence and spread of associated epidemics. Comprehensive and continuous research on the ST34-type *S.* Typhimurium with multicellular behavior is essential for its effective prevention, control, and for public health safety.

## Data Availability Statement

The original contributions presented in the study are included in the article/supplementary material, further inquiries can be directed to the corresponding authors.

## Ethics Statement

In this study, all strains from human and animal sources food were preserved in the laboratory, which did not involve the isolation and identification of bacteria from samples of relevant sources. It did not include animal research and human research, and there were no ethical issues related to living animals and human.

## Author Contributions

KC: methodology, data curation, and writing (original draft preparation). YG: validation and investigation. LL: supervision and resources. WZ, JL, ZZ, HH, and ZC: investigation. ML: supervision and project administration. JZ: conceptualization and writing (reviewing and editing). All authors contributed to the article and approved the submitted version.

## Funding

This work was supported by the National Natural Science Foundation of China (grant number 31972762); Guangdong Basic and Applied Basic Research Foundation (grant number 2021A1515010815); Walmart Foundation (grant numbers SA1703162, 61626817, and 52514289); National Broiler Industry Technology System Project (grant number cARS-41-G16); and Special Funds for the Cultivation of Guangdong College Students’ Scientific and Technological Innovation (grant number pdjh2021a0071).

## Conflict of Interest

The authors declare that the research was conducted in the absence of any commercial or financial relationships that could be construed as a potential conflict of interest.

## Publisher’s Note

All claims expressed in this article are solely those of the authors and do not necessarily represent those of their affiliated organizations, or those of the publisher, the editors and the reviewers. Any product that may be evaluated in this article, or claim that may be made by its manufacturer, is not guaranteed or endorsed by the publisher.

## Footnotes

^1^ http://enterobase.warwick.ac.uk/species/index/senterica

^2^ http://enterobase.Warwick.ac.uk/species/index/senterica
